# *Leishmania donovani* promastigotes evade the antimicrobial activity of neutrophil extracellular traps

**Published:** 2011-01-10

**Authors:** Christelle Gabriel, Robert W McMaster, Denis Girard, Albert Descoteaux

**Affiliations:** 1 INRS- Institut Armand-Frappier, Laval, Québec, Canada H7V 1B7; 2 Department of Medical Genetics, University of British Columbia, Vancouver, BC, Canada V6H 3Z6

## 

Upon their recruitment to a site of infection and their subsequent activation, neutrophils release DNA and a subset of their granule content to form filamentous structures, known as neutrophil extracellular traps, which capture and kill microorganisms. In this study, we show that *Leishmania* promastigotes induced the rapid release of neutrophil extracellular traps from human neutrophils and were trapped by these structures. The use of *Leishmania* mutants defective in the biosynthesis of either lipophosphoglycan or GP63 revealed that these two major surface promastigote virulence determinants were not responsible for inducing the release of neutrophil extracellular traps. We also demonstrate that this induction was independent of superoxide production by neutrophils. Finally, in contrast to wild type *L. donovani* promastigotes, mutants defective in lipophosphoglycan biosynthesis were highly susceptible to the antimicrobial activity of neutrophil extracellular traps. Altogether, our data suggest that neutrophil extracellular traps may contribute to the containment of *L. donovani* promastigotes at the site of inoculation, thereby facilitating their uptake by mononuclear phagocytes.

